# Feasibility of smart ring-based remote monitoring in hospitalized patients: evaluation of signal transmission latency and data acquisition continuity

**DOI:** 10.3389/fbioe.2025.1689794

**Published:** 2025-12-16

**Authors:** Hyunsun Lim, Jung Mi Han, Min Hyun Park, Joo Youn Kim, Haeyong Pak, Seung Jin Oh

**Affiliations:** 1 Department of Research and Analysis, National Health Insurance Service Ilsan Hospital, Goyang, Republic of Korea; 2 Smart Hospital Innovation Department, National Health Insurance Service Ilsan Hospital, Goyang, Republic of Korea; 3 Cardiovascular Center, National Health Insurance Service Ilsan Hospital, Goyang, Republic of Korea

**Keywords:** wearable device, smart ring, transmission latency, signal acquisition rate, vital sign

## Abstract

**Background:**

General wards rely on intermittent vital-sign checks, creating blind spots for deterioration detection under staffing constraints. While wearable sensors promise continuous ward-scale monitoring, real-world feasibility remains uncertain because prior studies emphasized signal accuracy rather than the timeliness and low-latency continuous monitoring system. This study addresses that gap by quantifying transmission latency and signal acquisition rate (SAR) of a ring-based system in routine surgical-ward care.

**Methods:**

We conducted a single-center retrospective analysis of 128 adult postoperative inpatients monitored with a finger-worn smart ring (CART-1 plus) from 23 December 2024 to 20 July 2025. Signals streamed via BLE to a dedicated receiver and hospital Wi-Fi. Primary endpoints were (i) transmission latency (device timestamp→server receipt) and (ii) SAR. Cached burst uploads at 0–1-s intervals were excluded from latency analyses. Prespecified acceptability thresholds were ≤10 min for BP latency, ≤5 min for SpO_2_ latency, and ≥80% for SAR. Associations with patient/environmental factors (mobility status, room type, time of day, network environment) were modeled.

**Results:**

Mean transmission latency was 7.23 min (BP) and 4.35 min (SpO_2_), both within predefined thresholds (10 and 5 min, respectively; p < 0.001). The mean SAR was 90.17% for BP and 90.62% for SpO_2_, significantly exceeding the 80% threshold (p < 0.001). Transmission latency was significantly lower in patients requiring no or full assistance with mobility, in those residing in single or double rooms (vs. quad), and during daytime monitoring (p < 0.001). For SpO_2_, latency was affected by mobility and change in network environment but not by room type or monitoring time. SAR was not significantly influenced by clinical variables but was higher after change in network environment (p < 0.05).

**Conclusion:**

In real-world surgical wards, a finger-worn ring achieved clinically acceptable transmission latency and high continuity, supporting feasibility for a low-latency continuous monitoring system. Findings identify modifiable environmental and patient-level factors that influence latency and can guide scale-up and integration with early-warning workflows.

## Introduction

In recent years, the need for continuous and non-invasive monitoring of hospitalized patients has grown significantly due to increasing patient acuity and staffing shortages. Traditional vital sign monitoring in general wards often relies on intermittent manual measurements by nursing staff, which may lead to missed early signs of deterioration, particularly during off-hours or in high-patient-volume settings (Rowland et al., 2024; Eddahchouri et al., 2022; Brown et al., 2014). In response, wearable medical technologies have emerged as promising alternatives that allow for automated, real-time physiological monitoring (Rowland et al., 2024; Eddahchouri et al., 2022; Breteler et al., 2018; Taenzer et al., 2010; Joung et al., 2023; Kwon et al., 2019; Kwon et al., 2022; Weenk et al., 2017). Among these technologies, smart rings—wearable devices worn on the finger—offer several advantages: they are minimally intrusive, comfortable for long-term wear, and capable of continuously measuring multiple physiological signals such as blood pressure and peripheral oxygen saturation (SpO_2_). Unlike wrist-worn devices, smart rings benefit from anatomical positioning closer to arteries and capillaries, potentially enhancing signal accuracy (van Rossum et al., 2022; Svensson et al., 2024; Cao et al., 2022; Kim et al., 2024; Briosa et al., 2025; van Steijn et al., 2025). Despite these advantages, practical implementation of smart rings in inpatient settings requires validation of two key aspects: (1) the timeliness of data transmission to the hospital server, and (2) the continuity and completeness of signal acquisition over time. These metrics are especially critical for detecting acute patient deterioration and integrating wearable data into clinical workflows (van Rossum et al., 2022). This study aims to evaluate the clinical feasibility of a smart ring-based monitoring system in general ward inpatients by analyzing (a) the signal transmission latency and (b) the signal acquisition rate (SAR). Prior work has established the architectural building blocks for real-time remote patient monitoring (RPM) in hospitals and highlighted the decisive role of network design in achieving clinically useful responsiveness, yet standardized ward-level benchmarks for latency and continuous data availability remain inconsistent, especially for ring-based wearables (Rowland et al., 2024; Eddahchouri et al., 2022; Breteler et al., 2018; Taenzer et al., 2010; Brown et al., 2014; Leenen et al., 2024; Buttazzo, 2011). Building on these insights, our study aims to achieve three practical advances: (1) we pre-specify clinically acceptable thresholds for a ward-level low-latency continuous monitoring system (BP latency ≤10 min; SpO_2_ latency ≤5 min; SAR ≥80%) and formally test whether a smart-ring system meets them; (2) we quantify the causal impact of network augmentation (denser access-points and optimized roaming) on latency and SAR under covariate adjustment, demonstrating infrastructure as a modifiable lever; and (3) we operationalize the feasibility of a low-latency continuous monitoring system by precisely defining latency, excluding batch-upload artifacts, and reporting prespecified subgroup and sensitivity analyses to guide deployment in general wards.

## Methods

### Study design and setting

This retrospective observational study was conducted at a tertiary general hospital as part of the institutional project titled “Development of Non-Face-to-Face Medical Service Using Wearable Devices (Smart Ring).” The study evaluated the feasibility and performance of a smart ring-based remote monitoring system for inpatients. Data were collected from 23 December 2024 to 20 July 2025. All procedures were approved by the Institutional Review Board of NHIS Ilsan Hospital (NHIMC-2025-06-033) and conducted in accordance with the Declaration of Helsinki.

### Study population

**FIGURE 1:**
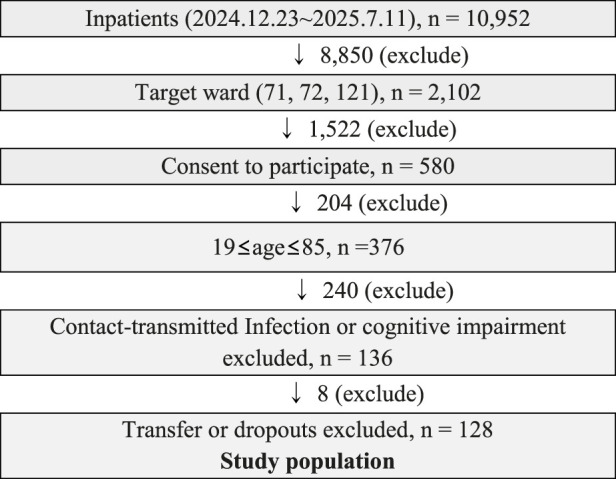
Flowchart.

Eligible participants were monitored postoperatively using the smart ring device. Patients admitted to the target wards (71, 72, and 121) between 23 December 2024, and 11 July 2025, were included if they were 19–85 years of age and provided informed consent. Patients were excluded if they were diagnosed with delirium during hospitalization or transferred to another unit during the monitoring period. Total of 128 patients met these criteria and included in the final analysis (Figure 1). As this was an exploratory study, no *a priori* sample size calculation was performed.

### Wearable device specification

The wearable device used in this study was CART-I plus (Sky Labs, Seongnam, Republic of Korea), a commercially available smart ring certified as a medical device by the Korean Ministry of Food and Drug Safety (MFDS). CART-I plus is a ring-type wearable designed for continuous, non-invasive cardiovascular monitoring in ambulatory and inpatient settings. The device integrates a photoplethysmography (PPG) sensor and 3-axis accelerometer to collect multi-parameter physiological signals. Blood pressure (BP) is estimated using a cuffless PPG-based algorithm, which analyzes pulse waveforms and applies machine learning–based regression models calibrated to initial reference measurements. The device is worn on the finger, allowing close contact with the peripheral vasculature, thereby improving signal fidelity compared to wrist-worn devices. CART-I plus continuously collects data and transmits it via Bluetooth Low Energy (BLE) to a dedicated smartphone application, which uploads the data to a hospital server in a low-latency continuous monitoring system.

### Network architecture for signal transmission

**FIGURE 2:**
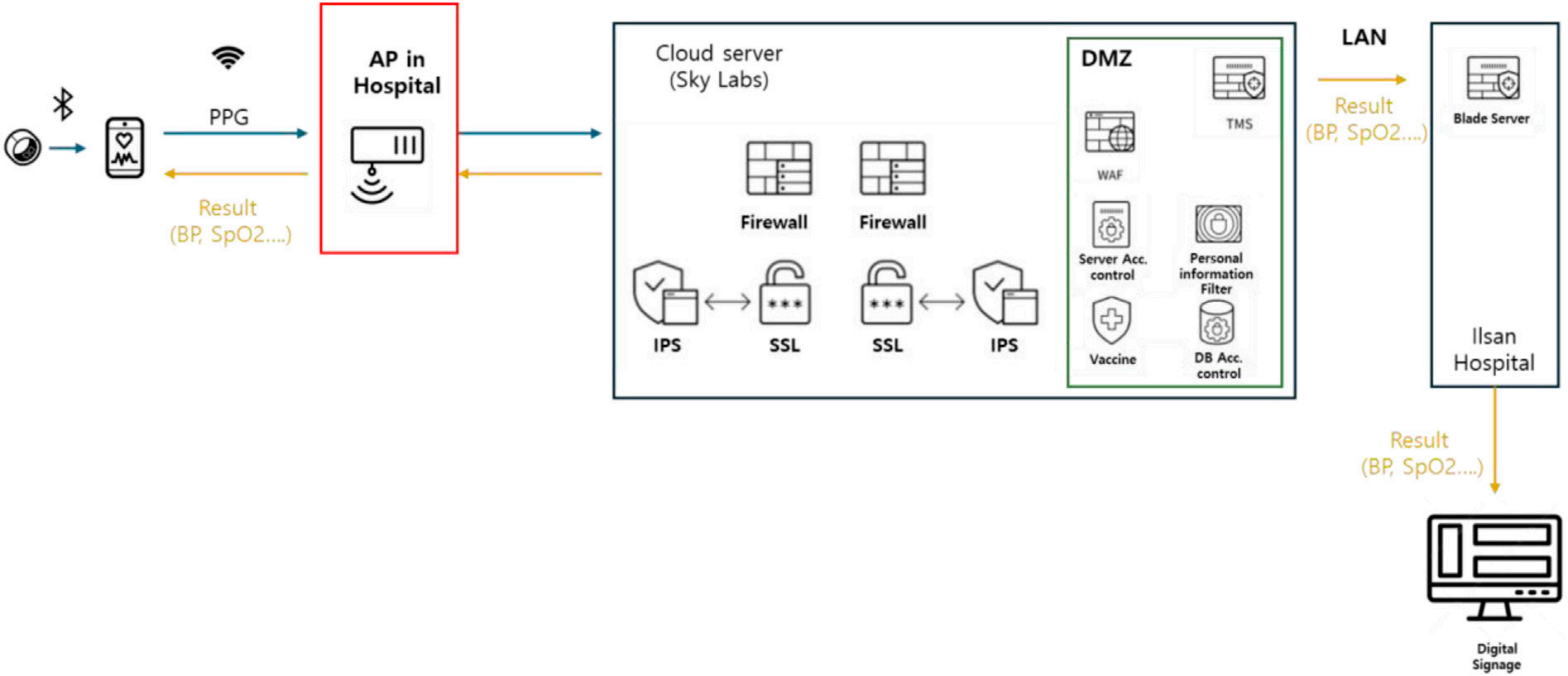
Network architecture schematic (device→receiver→AP→server).

In this study, the smart ring (CART-I plus) transmitted physiological data via Bluetooth Low Energy (BLE) to a dedicated receiver device assigned to each patient. The dedicated receiver assigned to each patient was implemented using a Samsung Galaxy A25 5G smartphone (Samsung Electronics, Suwon, Republic of Korea). The device operates on the Android OS and supports Bluetooth Low Energy (BLE) 5.3 for communication with the smart ring and dual-band Wi-Fi (2.4 GHz/5 GHz) for data relay to the hospital network. It is equipped with an Exynos 1,280 processor, 6 GB RAM, and a 5,000 mAh battery, ensuring stable operation throughout continuous monitoring. In this study, the smartphone functioned as a dedicated gateway, facilitating one-to-one pairing with the patient’s hospital ID, maintaining local buffering during temporary disconnections, and automatically resuming encrypted data transmission to the on-premise server once connectivity was restored. Each receiver relayed the data to the on-premise hospital server using the hospital’s wireless infrastructure. Specifically, wireless access points (APs) were installed on the ward ceilings at 10-m intervals, forming a dense Wi-Fi network to ensure robust connectivity throughout the monitoring area. The APs were optimized for continuous data collection and high-traffic environments, minimizing data loss and latency due to coverage gaps or handover delays. This architecture allowed for a low-latency continuous monitoring system and ensured uninterrupted signal flow from the wearable device to the hospital server, even as patients moved within the ward. Locally cached data on the receiver were automatically uploaded when temporary disconnections occurred, preserving the continuity and integrity of the physiological signal record. Communication between the NHIS Ilsan Hospital and the private cloud is allowed only through a fixed IP address via the proxy server, while all other external connections are blocked by the cloud-side firewall. Within the private cloud, the system is deployed in the public zone, and all communication channels are encrypted through an IPsec VPN tunnel. Data transmission between the hospital and the private cloud is secured using both VPN and proxy server encryption mechanisms. Access to the proxy server is restricted to authorized connections only, enforced by firewall policy configurations (Figure 2).

### Wearable device monitoring and data collection

**FIGURE 3:**
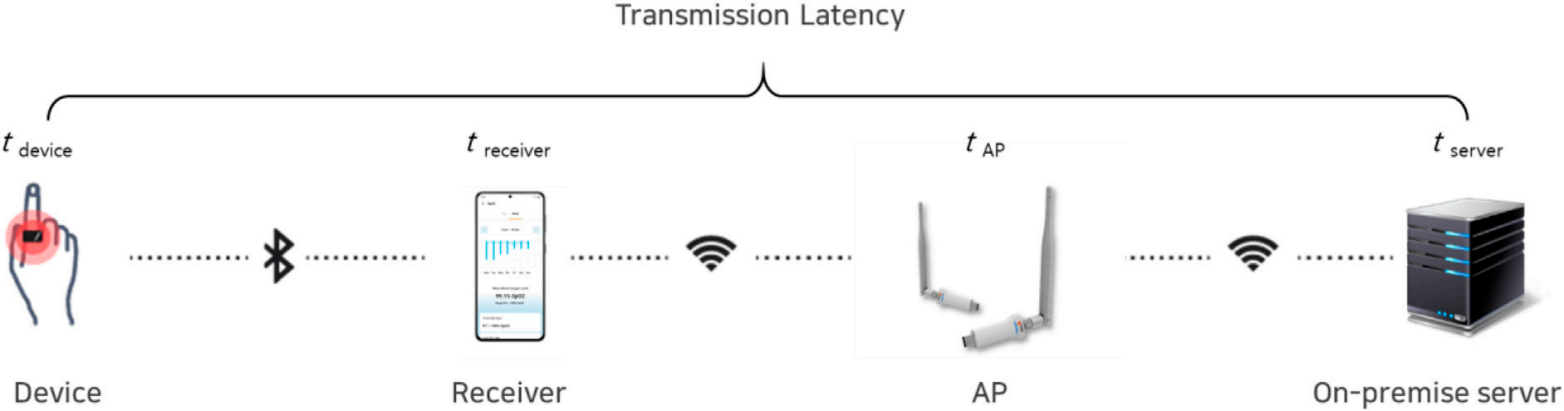
Latency definition and temporal diagram.

Smart rings were applied immediately after surgery and worn continuously until discharge. The device recorded physiological signals including blood pressure (BP) and peripheral oxygen saturation (SpO_2_), and transmitted the data to the hospital server via wireless communication. Two core performance metrics were evaluated:

Transmission latency: Defined as the time difference (in minutes) between the signal acquisition timestamp recorded by the device and the timestamp of successful reception by the hospital server (Figure 3).

$$\text{Transmission latency}=\left(\text{server receipt timestamp}\;-\text{device acquisition timestamp}\right)$$

Signal acquisition rate (SAR): Defined as the percentage of successfully transmitted data points relative to the total expected data points over the monitoring period.

$$\text{Signal acquisition rate }\left(\text{SAR},\%\right)=\left(\frac{Duration\;of\;valid\;signal\;acquisition\;\left(n\right)}{Total\;monitoring\;duration\;\left(n\right)}\right)\times 100$$

These metrics were used to evaluate both the timeliness and completeness of data acquisition and transmission, thereby reflecting the feasibility and reliability of the smart ring-based remote monitoring system in a real-world clinical setting. In this study, the low-latency continuous monitoring system denotes an operational state in which end-to-end data arrival at the hospital server occurs within ≤10 min for BP and ≤5 min for SpO_2_ from on-device acquisition (van Rossum et al., 2022). This differs from hard real-time systems that guarantee deterministic worst-case deadlines; our definition reflects clinically acceptable responsiveness for ward-level remote monitoring. To ensure the validity of real-time RPM, locally cached bulk-transmitted data (i.e., data uploaded with 0–1 s intervals following server reconnection) were excluded from the latency analysis. Device removal was defined as an interval exceeding 1 h between data points based on local timestamps.

### Exposure variables

To evaluate the factors influencing transmission latency and SAR, the following variables were analyzed: mobility level (independence, partial assistance, or full assistance), room type (single, double, or quad occupancy), change in network environment (Unaugmented setup vs. augmented setup), and time of data acquisition (daytime vs. nighttime). In the network environment, latency was mainly due to structural limitations before the Wi-Fi upgrade and weak signal strength in some room locations. Accordingly, the network environment was defined as unaugmented (before upgrade) and augmented (after upgrade). Additional covariates included age, gender, use of orthopedic brace, length of hospital stay, number of surgery days, and nursing acuity score.

### Statistical analysis

Data from descriptive analyses were summarized using mean ± standard deviation, number, or proportions. A one-sample t-test was used to determine whether the mean transmission latency was significantly lower than the predefined thresholds (e.g., 1, 5, 10, 15 min, etc.) (Breteler et al., 2018; Kroll et al., 2016; Hur et al., 2020), and whether the signal acquisition rate (SAR) exceeded the threshold of 70%, 80%, or 90% (Breteler et al., 2018; Kroll et al., 2016; Neal et al., 2024). The association of exposures with the outcome was examined using general linear model with adjustment for confounding variables including gender, age, use of orthopedic brace, mobility level, room type, length of hospital stay, number of surgery days, and nursing acuity score. All analyses were performed using SAS, version 9.4 (SAS Institute, Cary, NC, United States) (SAS Institute Inc., 2013). P-value of <0.05 was considered to be statistically significant.

## Results

### Participant characteristics

A total of 128 inpatients were included in the final analysis. The mean age was 51.7 years (SD: 12.4), and 76 patients (59.38%) were female. Most participants were in their 50s (27.34%) or 60s (26.56%), with age ranging from 20 to over 80 years. The average length of hospital stay was 6.81 days, and the mean number of surgery days was 4.0. Approximately 39.8% of participants exhibited some degree of movement restriction. Among these, 43 patients (33.59%) required partial assistance with mobility, and 8 patients (6.25%) required full assistance. The average nursing acuity score was 20.26, with 90.6% of patients classified as Level 2 or 3 in terms of nursing care needs. Regarding room type, 106 patients (82.81%) were admitted to quad occupancy, while 10 (7.81%) and 12 (9.38%) were placed in single and double occupancy, respectively. In terms of change in network environment, 41 cases (32.03%) occurred under the unaugmented setup, and 87 cases (67.97%) under the augmented setup. The mean duration of device use was 6.81 days, consistent with the average length of hospital stay (Table 1).

**TABLE 1:** Demographic characteristics of the study cohorts.

Variables	n (%)
Overall	128 (100.00)
Gender	
Male	52 (40.63)
Female	76 (59.38)
Age (years)	51.70 ± 12.40^a^
Age	
20–29	7 (5.47)
30–39	17 (13.28)
40–49	30 (23.44)
50–59	35 (27.34)
60–69	34 (26.56)
70–79	4 (3.13)
≥80	1 (0.78)
Length of hospital stay	6.81 ± 7.81^a^
Number of surgery days	4.00 ± 3.90^a^
Mobility level	
Independence	77 (60.16)
Partial assistance	43 (33.59)
Full assistance	8 (6.25)
Nursing acuity score (KPCS)	20.26 ± 7.08^a^
Reclassification of nursing acuity	
1	0 (0.00)
2	74 (57.81)
3	42 (32.81)
4	12 (9.38)
Wearing period (days)	6.81 ± 3.87^a^
Room type	
Single occupancy	10 (7.81)
Double occupancy	12 (9.38)
Quad occupancy	106 (82.81)
Change in network environment	
Unaugmented setup	41 (32.03)
Augmented setup	87 (67.97)

^a^ Mean ± SD; SD, standard deviation; KPCS, korean patient classification system.

### Transmission latency and signal acquisition rate

**FIGURE 4:**
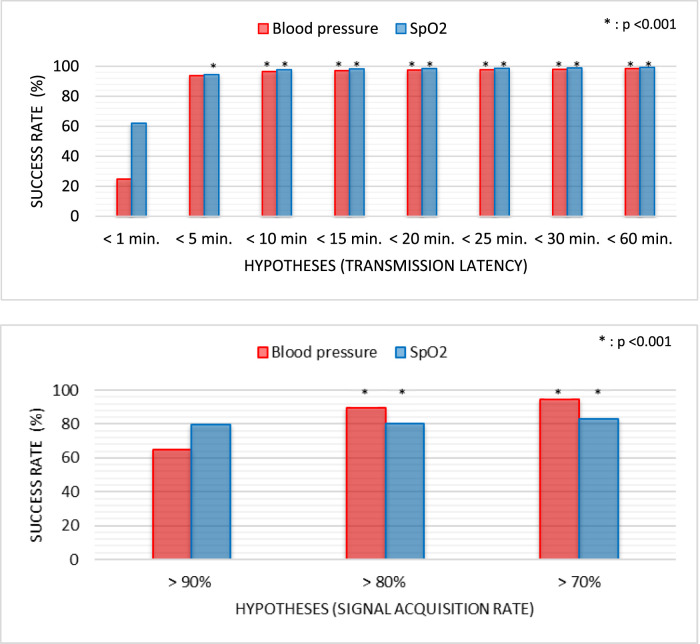
Comparison of transmission latency and signal acquisition rate across statistical hypotheses. min, Minutes.

The mean transmission latency was 7.23 min for blood pressure (BP) signals and 4.35 min for peripheral oxygen saturation (SpO_2_). These values were significantly below the predefined thresholds of 10 min (BP) and 5 min (SpO_2_), respectively (both p < 0.001). The signal acquisition rate (SAR) was 90.17% for BP and 90.62% for SpO_2_. Both exceeded the predefined acceptability threshold of 80% with statistical significance (p < 0.001), indicating high continuity and completeness of physiological data collection across the monitoring period (Figure 4; Supplementary Table S1).

### Factors influencing transmission latency and signal acquisition rate

**FIGURE 5:**
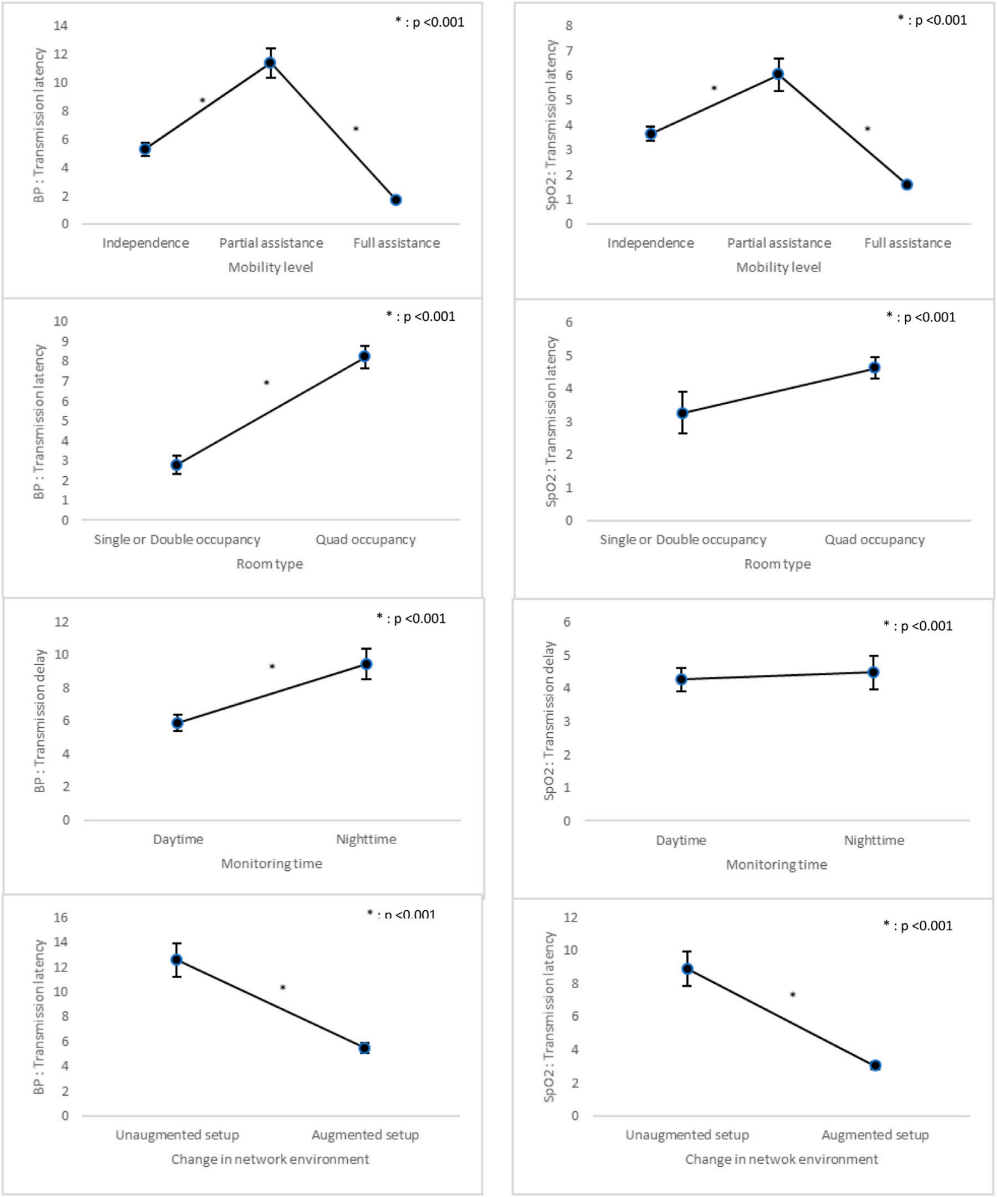
Comparison of Transmission latency Across Variables. p-value: Adjusted with Gender, Age, Use of orthopedic brace, Mobility level, Room type, Length of Hospital Stay, Number of surgery days, and Nursing acuity score; BP: Blood Pressure.

Transmission latency for BP was significantly associated with the primary exposure after adjustment for confounding variables. Patients who either required no assistance or full assistance with mobility exhibited shorter latency compared to those requiring partial assistance (p < 0.001). Similarly, patients in single or double occupancy had significantly lower latency than those in quad occupancy (p < 0.001), and signals acquired during daytime had shorter delays than those obtained at night (p < 0.001). Additionally, under the augmented network setup, signals showed a significant reduction in latency (p < 0.001). For SpO_2_, transmission latency was also lower in patients with no or full mobility assistance (p < 0.001) and under the augmented network setup (p < 0.001). However, there were no statistically significant differences by room type or monitoring time (p = 0.107) (Figure 5; Supplementary Table S2).

**FIGURE 6:**
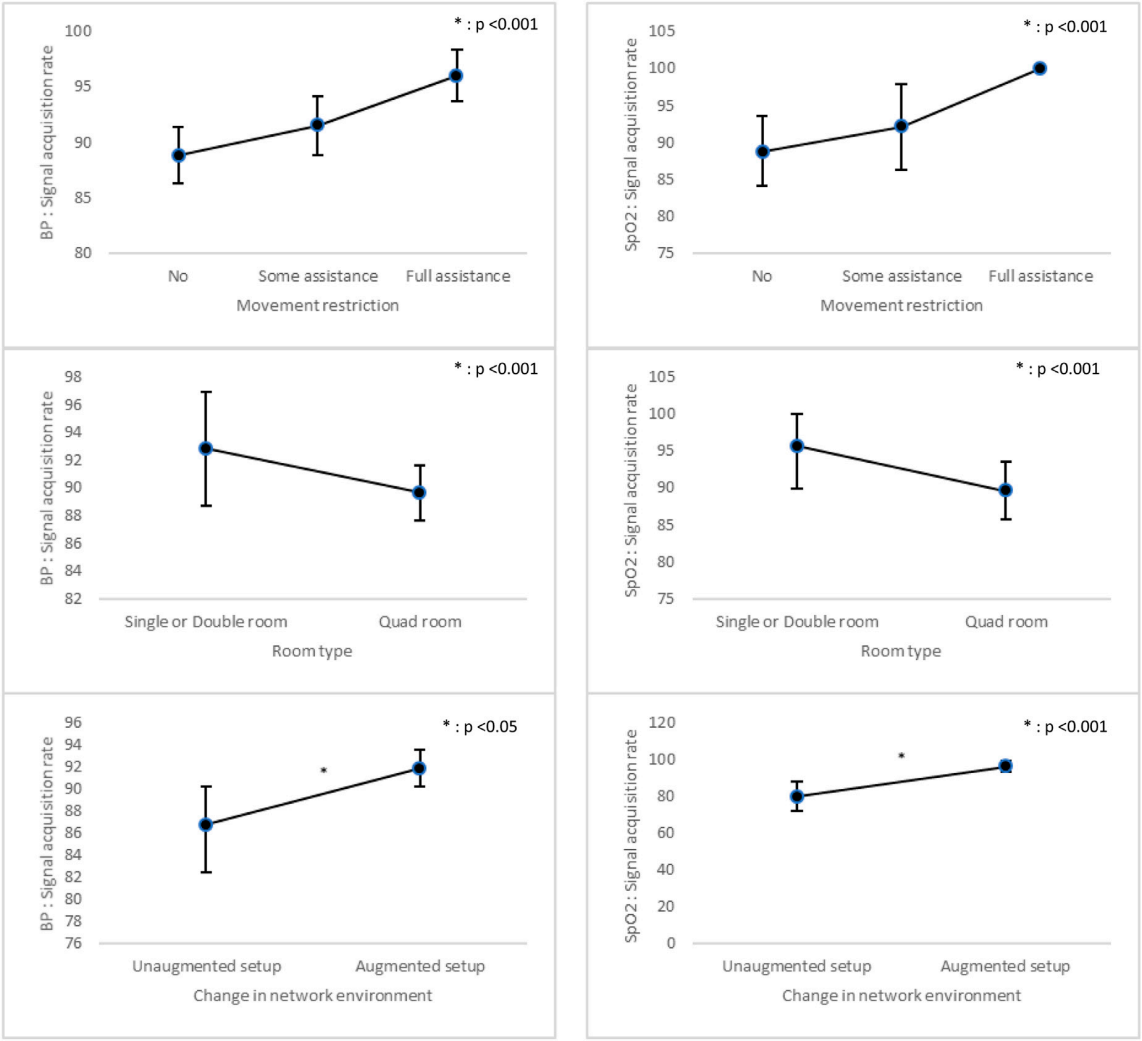
Comparison of Signal acquisition rate Across Variables. p-value: Adjusted with Gender, Age, Use of orthopedic brace, Movement restriction, Room type, Length of Stay, Days with surgery, and Nursing acuity; BP: Blood Pressure.

SAR was generally stable across subgroups but showed a significant improvement under the augmented network setup, for both BP (p = 0.036) and SpO_2_ (p < 0.001). No other clinical or environmental factors—including mobility level, room type, or collection time—demonstrated significant associations with SAR (Figure 6; Supplementary Table S2).

### Sensitivity analysis

Sensitivity analyses were conducted to evaluate the robustness of findings across various subgroups, including gender, age (<60 vs. ≥60 years), nursing acuity level (Level 1–2 vs. Level 3–4), number of surgery days (<2 vs. ≥2), and length of hospital stay (<5 days vs. ≥5 days).

**FIGURE 7:**
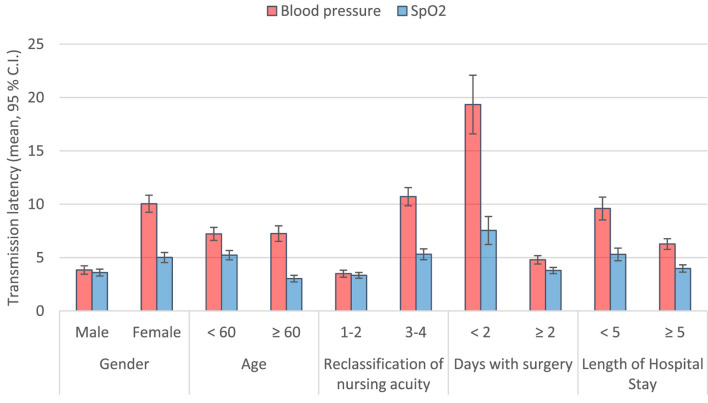
Comparison of Transmission latency by Subgroup variables.

For blood pressure, transmission latency differed significantly across all subgroup comparisons (p < 0.001 for each) after adjustment for confounding variables. For SpO_2_, significant differences in latency were observed according to age, nursing acuity level, and length of hospital stay (all p < 0.001), but not by gender or days with surgery. No significant differences in SAR were found in any subgroup analysis (Supplementary Table S3; Figure 7).

## Discussion

This study demonstrated that a smart ring–based remote monitoring system can achieve clinically acceptable transmission latency and high signal acquisition continuity in inpatients. The mean transmission latency was 7.23 min for blood pressure (BP) and 4.35 min for peripheral oxygen saturation (SpO_2_), both within predefined performance thresholds. Signal acquisition rates exceeded 90% for both parameters, indicating robust data continuity throughout the monitoring period.

While wearable devices have been widely studied for RPM in ambulatory or post-discharge settings, relatively few studies have evaluated their applicability in real-world inpatient environments. Downey et al. reported that continuous monitoring with chest-worn sensors improved detection of clinical deterioration in general wards, although false alarms due to motion artifacts and poor adherence were noted (Downey et al., 2018). Similarly, Breteler et al. demonstrated that wireless vital sign monitoring improved early sepsis detection in postoperative patients (Breteler et al., 2018). These prior studies highlight the need for wearable RPM systems that are both accurate and timely, particularly in higher-acuity settings (Lee et al., 2024; Shin and Shin, 2024). In this context, low transmission latency is essential to ensure that clinicians receive actionable information in near real time, while high signal acquisition rates (SAR) are critical for maintaining continuous physiologic data streams and enabling the detection of subtle changes in patient status. Failure to meet minimum thresholds for either metric can compromise the clinical utility of RPM systems.

Notably, few existing studies have treated latency and SAR as primary quantitative endpoints. Studies by Breteler et al. and Kroll et al. have considered latency ≤5 min and data availability ≥80% as clinically acceptable standards for inpatient monitoring (Breteler et al., 2018; Kroll et al., 2016; Hur et al., 2020; Neal et al., 2024). In comparison, our findings with a ring-type device (CART-I plus) demonstrated not only compliance with these standards, but also superior performance in signal acquisition.

Moreover, our analysis identified patient-level and environmental factors that influence latency—insights often lacking in prior research. Among these, mobility level had a significant impact. Patients requiring partial assistance exhibited higher latency than those who were either fully independent or fully dependent. This U-shaped relationship may be explained by movement artifacts, a known limitation in wearable sensing. Partially mobile patients may move intermittently or reposition frequently, disrupting signal stability and delaying transmission. This observation aligns with earlier reports describing degraded signal quality due to patient movement (Svensson et al., 2024; Cao et al., 2022).

In addition, room type and days with surgery were associated with latency. Patients in quad rooms showed longer transmission delays compared to those in single or double occupancy, possibly due to network congestion or wireless interference in multi-bed environments. Similarly, latency was lower during daytime hours, likely reflecting stronger network performance and more stable infrastructure availability. As a desirable case, a 64-year-old postoperative patient in a single room was able to walk independently but spent most of the time resting in bed. Research staff inspected the device twice daily for battery level and network connection, adjusting the sensor when necessary. Blood pressure and oxygen saturation data were transmitted to the server within about 1 min on average, and the signal acquisition rate (SAR) was consistently maintained above 95%. As a delayed case, a 49-year-old female patient immediately after surgery was admitted to a four-bed room and required partial assistance for mobility (nursing acuity level 2). Before the Wi-Fi upgrade, the Bluetooth connection between the receiver and the smart ring remained active, but data from specific time intervals failed to transmit to the server. The research team switched the communication mode to 5G and readjusted the sensor placement. Transmission delays of approximately 20 min for blood pressure and 23 min for oxygen saturation were observed, after which normal data flow was restored. These findings are consistent with Kroll et al., who emphasized the role of network infrastructure readiness in enabling reliable RPM (Rowland et al., 2024; van Noort et al., 2024).

Smart ring systems such as CART-I plus rely on Bluetooth communication with a receiver device, which then transmits data to hospital servers via Wi-Fi. As such, network topology and signal interference become key determinants of data transmission quality. Unlike hardwired ICU monitors, wearable devices operate within decentralized wireless ecosystems, making them inherently more vulnerable to environmental conditions.

Interestingly, while latency was sensitive to both patient and environmental factors, SAR remained relatively stable across all subgroups. A significant improvement in SAR was observed only under the augmented network setup, suggesting that a system upgrade or protocol adjustment may have enhanced server connectivity or data integrity at that point.

Taken together, these findings suggest that smart rings can serve as reliable tools for continuous physiologic monitoring in general ward settings. By maintaining high SAR and low latency under real-world conditions, they may support earlier recognition of clinical deterioration without requiring invasive or labor-intensive monitoring.

However, several limitations should be acknowledged. This study was conducted at a single tertiary general hospital, and findings may not generalize to other institutions with different physical layouts or network infrastructures. Additionally, while latency levels were acceptable for ward monitoring, they may not meet the real-time demands of high-acuity environments such as ICUs. Finally, although this study focused on technical feasibility, clinical outcome measures such as reduction in adverse events or response time were not assessed.

Future studies should aim to integrate these systems with predictive algorithms and alert platforms to maximize their impact on patient safety and clinical workflow.

## Conclusion

This study demonstrated that a smart ring–based remote monitoring system can achieve clinically acceptable performance in a real-world inpatient general ward setting. The system exhibited low transmission latency and high signal acquisition rate (SAR), meeting commonly accepted thresholds for continuous physiological monitoring. Moreover, transmission latency was influenced by patient mobility, room type, and monitoring time, suggesting that both clinical and environmental factors must be considered when implementing wearable-based monitoring at scale.

These findings support the feasibility of deploying wearable devices for low-latency continuous monitoring system in general hospital wards. Importantly, they provide a foundation for future research aimed at leveraging continuous wearable data for predictive analytics, early warning systems, and improved clinical outcomes.

As healthcare systems continue to seek scalable, non-invasive monitoring solutions, smart rings may offer a promising balance of usability, accuracy, and integration potential. Further studies incorporating outcome-based validation and AI-powered early detection models will be essential to maximize the clinical value of wearable-based remote patient monitoring.

## Data Availability

The datasets presented in this article are not readily available due to the hospital’s data security policy. Requests to access the datasets should be directed to HL, hsunlim@nhimc.or.kr.
