# Supplementary material for: Feasibility of smart ring-based remote monitoring in hospitalized patients: evaluation of signal transmission latency and data acquisition continuity
Source: Front Bioeng Biotechnol. 2025 Dec 16;13:1689794. doi: 10.3389/fbioe.2025.1689794 (PMC12750146; doi:10.3389/fbioe.2025.1689794)
Supplement: Supplementary file 1 [file Table1.docx]

Comparison of transmission latency and Signal Acquisition rate across statistical hypotheses

| Outcome | Hypotheses | Blood pressure | | | | | | SpO2 | | | | | | | | |  |
| --- | --- | --- | --- | --- | --- | --- | --- | --- | --- | --- | --- | --- | --- | --- | --- | --- | --- |
|  |  | n | % | | *p-value* | | | n | | % | | | *p-value* | | |  |  |
| Transmission latency | | 47,019 | | 7.23±51.81* | |  | | | 62,168 | | 4.35±37.27* | | |  | | | |
|  | < 1 min. | 11,665 | 24.81 | | 1.000 | | | 38,572 | | 62.04 | | | 1.000 | | |  |  |
|  | < 5 min. | 44,102 | 93.80 | | 1.000 | | | 58,746 | | 94.50 | | | <0.001 | | |  |  |
|  | < 10 min | 45,375 | 96.50 | | <0.001 | | | 60,759 | | 97.73 | | | <0.001 | | |  |  |
|  | < 15 min. | 45,654 | 97.10 | | <0.001 | | | 61,079 | | 98.25 | | | <0.001 | | |  |  |
|  | < 20 min. | 45,829 | 97.47 | | <0.001 | | | 61,249 | | 98.52 | | | <0.001 | | |  |  |
|  | < 25 min. | 45,961 | 97.75 | | <0.001 | | | 61,355 | | 98.69 | | | <0.001 | | |  |  |
|  | < 30 min. | 46,053 | 97.95 | | <0.001 | | | 61,463 | | 98.87 | | | <0.001 | | |  |  |
|  | < 60 min. | 46,319 | 98.51 | | <0.001 | | | 61,702 | | 99.25 | | | <0.001 | | |  |  |
| Signal Acquisition rate | | 128 | | 90.17±10.17* | |  | | | 128 | | 90.62±19.48* | | |  | | | |
|  | > 90% | 128 | 64.84 | | 0.423 | | | 128 | | 79.69 | | | 0.360 | | |  |  |
|  | > 80% | 128 | 89.84 | | <0.001 | | | 128 | | 80.47 | | | <0.001 | | |  |  |
|  | > 70% | 128 | 94.53 | | <0.001 | | | 128 | | 82.81 | | | <0.001 | | |  |  |
| * Mean±SD | |  | |  | | |  |  | | | |  | | |  | |  |

Comparison of Transmission latency and Signal Acquisition rate Across Variables

| Outcome | Variables | Blood pressure | | | SpO2 | | |
| --- | --- | --- | --- | --- | --- | --- | --- |
|  |  | n | Mean±SD | *p-value** | n | Mean±SD | *p-value** |
| Transmission latency | Overall | 47,019 | 7.23±51.81 |  | 62,168 | 4.35±37.27 |  |
|  | Movement restriction |  |  | <0.001 |  |  | <0.001 |
|  | No | 25,277 | 5.28±37.81 | 1 | 32,780 | 3.64±25.90 | 1 |
|  | Some assistance | 17,516 | 11.39±71.49 | 2 | 23,484 | 6.03±52.28 | 2 |
|  | Full assistance | 4,226 | 1.69±3.11 | 1 | 5,904 | 1.60±2.65 | 1 |
|  | Room type |  |  | <0.001 |  |  | 0.423 |
|  | Single or Double room | 8,594 | 2.76±21.78 |  | 12,265 | 3.27±35.47 |  |
|  | Quad room | 38,425 | 8.23±56.33 |  | 49,903 | 4.62±37.70 |  |
|  | Day/Night use |  |  | <0.001 |  |  | 0.107 |
|  | Day | 29,189 | 5.88±43.86 |  | 38,359 | 4.27±35.62 |  |
|  | Night | 17,830 | 9.44±62.63 |  | 23,809 | 4.49±39.78 |  |
|  | Period |  |  | <0.001 |  |  | <0.001 |
|  | < 2025.2.17 | 11,724 | 12.57±75.81 |  | 14,110 | 8.89±63.96 |  |
|  | ≥ 2025.2.17 | 35,295 | 5.46±40.67 |  | 48,058 | 3.02±24.25 |  |
| Signal Acquisition rate | Overall | 128 | 90.17±10.17 |  | 128 | 90.62±19.48 |  |
|  | Movement restriction |  |  | 0.098 |  |  | 0.294 |
|  | No | 77 | 88.84±11.21 |  | 77 | 88.80±20.58 |  |
|  | Some assistance | 43 | 91.48±8.54 |  | 43 | 92.13±18.86 |  |
|  | Full assistance | 8 | 95.98±2.79 |  | 8 | 100.00±0.00 |  |
|  | Room type |  |  | 0.915 |  |  | 0.992 |
|  | Single or Double room | 22 | 92.81±9.22 |  | 22 | 95.58±12.87 |  |
|  | Quad room | 106 | 89.63±10.32 |  | 106 | 89.59±20.48 |  |
|  | Period |  |  | 0.036 |  |  | <0.001 |
|  | < 2025.2.17 | 41 | 86.72±13.46 |  | 41 | 79.69±25.34 |  |
|  | ≥ 2025.2.17 | 87 | 91.80±7.76 |  | 87 | 95.77±13.34 |  |

* Adjusted with Sex, Age, Brace use, Movement restriction, Room type, Length of Stay, Days with surgery, and Nursing acuity; SD : Standard deviation

Comparison of Transmission latency and Signal Acquisition rate Across Variables : Sensitivity analysis

| Outcome | Variables | Blood pressure | | | SpO2 | | |
| --- | --- | --- | --- | --- | --- | --- | --- |
|  |  | n | Mean±SD | *p-value** | n | Mean±SD | *p-value** |
| Transmission latency | Gender |  |  |  |  |  |  |
|  | Male | 21,311 | 3.84±28.54 | <0.001 | 29,197 | 3.60±28.00 | 0.9731 |
|  | Female | 25,708 | 10.05±64.94 |  | 32,971 | 4.01±43.86 |  |
|  | Age |  |  | <0.001 |  |  | <0.001 |
|  | < 60 | 28,669 | 7.22±52.65 |  | 37,240 | 5.23±43.42 |  |
|  | ≥ 60 | 18,350 | 7.25±50.47 |  | 24,928 | 3.03±25.40 |  |
|  | Reclassification of nursing acuity |  |  | <0.001 |  |  | <0.001 |
|  | 1-2 | 22,655 | 3.49±25.39 |  | 30,281 | 3.34±24.49 |  |
|  | 3-4 | 24,364 | 10.71±67.50 |  | 31,887 | 5.31±46.22 |  |
|  | Days with surgery |  |  | <0.001 |  |  | 0.6676 |
|  | < 2 | 27,095 | 7.68±55.27 |  | 35,811 | 4.20±36.10 |  |
|  | ≥ 2 | 13,008 | 4.27±31.68 |  | 16,839 | 4.35±32.90 |  |
|  | Length of Stay |  |  | <0.001 |  |  | <0.001 |
|  | < 5 | 13,633 | 9.60±63.49 |  | 17,598 | 5.30±39.90 |  |
|  | ≥ 5 | 33,386 | 6.27±46.17 |  | 44,579 | 3.98±36.18 |  |
| Signal Acquisition rate | Gender |  |  | 0.711 |  |  | 0.868 |
|  | Male | 52 | 90.53±9.67 |  | 52 | 92.41±18.26 |  |
|  | Female | 76 | 89.93±10.56 |  | 76 | 89.39±20.30 |  |
|  | Age |  |  | 0.651 |  |  | 0.483 |
|  | < 60 | 89 | 89.20±11.22 |  | 89 | 88.00±21.52 |  |
|  | ≥ 60 | 39 | 92.40±6.87 |  | 39 | 96.59±11.95 |  |
|  | Reclassification of nursing acuity |  |  | 0.837 |  |  | 0.416 |
|  | 1-2 | 74 | 90.06±11.94 |  | 74 | 90.55±20.26 |  |
|  | 3-4 | 54 | 90.33±7.19 |  | 54 | 90.71±18.54 |  |
|  | Days with surgery |  |  | 0.489 |  |  | 0.687 |
|  | < 2 | 72 | 89.86±11.77 |  | 72 | 89.28±21.89 |  |
|  | ≥ 2 | 39 | 90.00±8.21 |  | 39 | 89.39±18.24 |  |
|  | Length of Stay |  |  | 0.685 |  |  | 0.185 |
|  | < 5 | 57 | 89.00±13.04 |  | 57 | 86.60±23.61 |  |
|  | ≥ 5 | 71 | 91.11±8.06 |  | 71 | 93.84±14.79 |  |

* Adjusted with Sex, Age, Brace use, Movement restriction, Room type, Length of Stay, Days with surgery, and Nursing acuity; SD : Standard deviation
